# Supplementary material for: Divergence of TORC1-mediated stress response leads to novel acquired stress resistance in a pathogenic yeast
Source: PLoS Pathog. 2023 Oct 23;19(10):e1011748. doi: 10.1371/journal.ppat.1011748 (PMC10621968; doi:10.1371/journal.ppat.1011748)
Supplement: S1 Fig — (A) Basal survival rates (r) at different H2O2 concentrations in C. glabrata and S. cerevisiae were quantified using Colony Forming Unit (CFU) ratios between cells treated with H2O2 and mock treated ones. The red dots and vertical lines show the mean and 95% confidence intervals based on 1000 bootstrap replicates. Individual data points are shown in different shapes grouped by the date of the experiment. (B) Acquired Stress Resistance (ASR) at different H2O2 secondary stress levels in the two species. ASR-score is defined as the fold increase in survival after the H2O2 treatment as a result of the primary stress (phosphate starvation). r’ is the survival rate with the primary stress and r is the same as in (A), i.e., without the primary stress. The red dots and lines have the same meanings as in (A). (C) ASR for H2O2 at different primary stress length. 100 mM and 10 mM of H2O2 were used as the secondary stress for the two species as in Fig 1. The dotted line shows an ASR score of 1 (i.e., no increase in survival due to the primary stress). A paired t-test was performed on the underlying survival rates (r and r’) for each of the six species-by-duration combinations. After Bonferroni correction, all three tests in C. glabrata had P < 0.05, while all three tests in S. cerevisiae yielded P > 0.5. (PDF) [file ppat.1011748.s001.pdf]

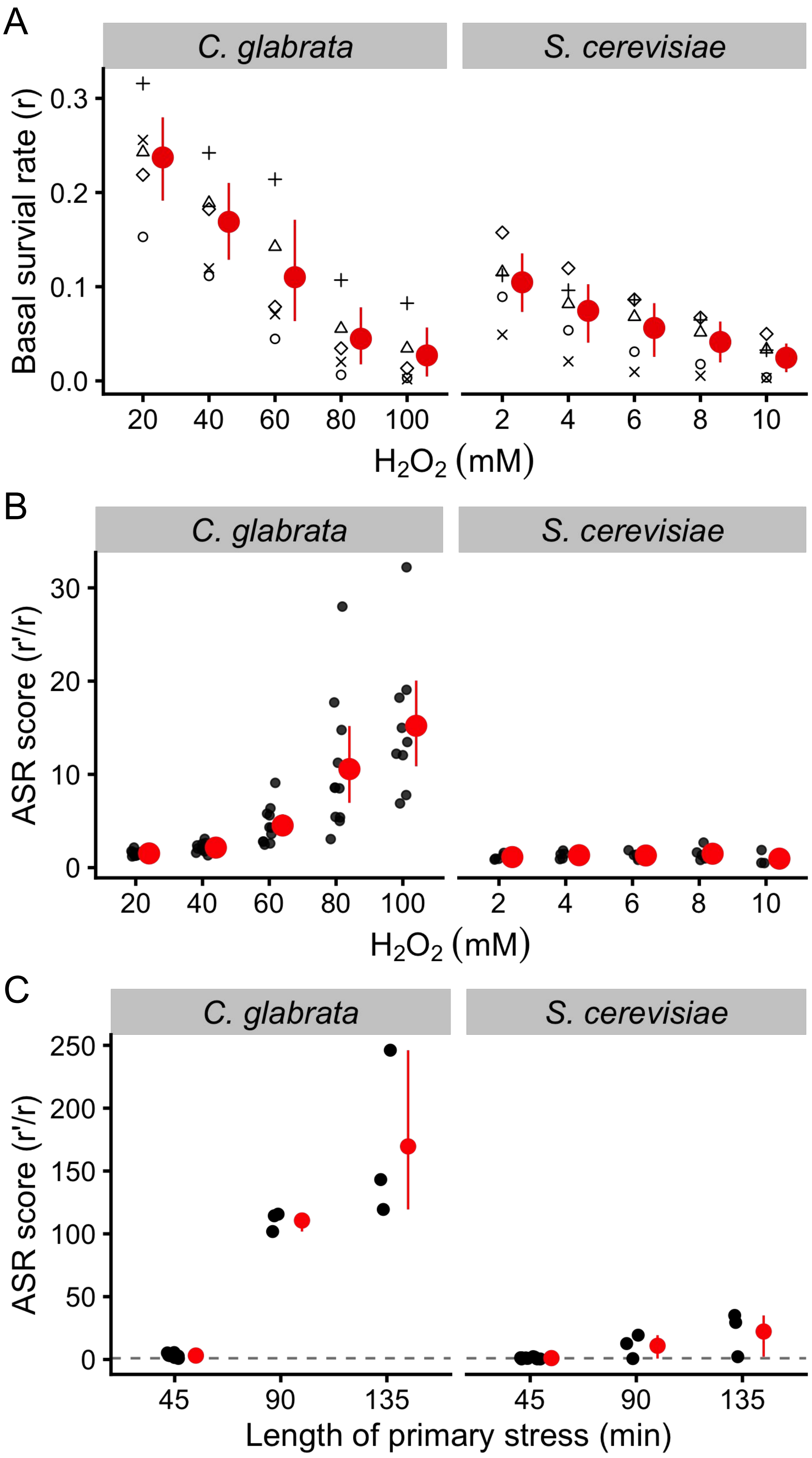

A
C. glabrata
S. cerevisiae
Basal survial rate (r)
0.3
0.2
0.1
0.0
20
40
60
80
100
2
4
6
8
10
H2O2 (mM)
B
C. glabrata
S. cerevisiae
ASR score (r'/r)
30
20
10
0
20
40
60
80
100
2
4
6
8
10
H2O2 (mM)
C
C. glabrata
S. cerevisiae
ASR score (r'/r)
250
200
150
100
50
0
45
90
135
45
90
135
Length of primary stress (min)

Supplementary Figure 1. Basal survival rates and phosphate starvation induced acquired resistance for H_2O_2 at different primary and secondary stress conditions. (A) Basal survival rates (r) at different H_2O_2 concentrations in *C. glabrata* and *S. cerevisiae* were quantified using Colony Forming Unit (CFU) ratios between cells treated with H_2O_2 and mock treated ones. The red dots and vertical lines show the mean and 95% confidence intervals based on 1000 bootstrap replicates. Individual data points are shown in different shapes grouped by the date of the experiment. (B) Acquired Stress Resistance (ASR) at different H_2O_2 secondary stress levels in the two species. ASR-score is defined as the fold increase in survival after the H_2O_2 treatment as a result of the primary stress (phosphate starvation). r' is the survival rate with the primary stress and r is the same as in (A), i.e., without the primary stress. The red dots and lines have the same meanings as in (A). (C) ASR for H_2O_2 at different primary stress length. 100 mM and 10 mM of H_2O_2 were used as the secondary stress for the two species as in Fig. 1. The dotted line shows an ASR score of 1 (i.e., no increase in survival due to the primary stress). A paired t-test was performed on the underlying survival rates (r and r') for each of the six species-by-duration combinations. After Bonferroni correction, all three tests in *C. glabrata* had $P < 0.05$, while all three tests in *S. cerevisiae* yielded $P > 0.5$
