# Supplementary material for: Divergence of TORC1-mediated stress response leads to novel acquired stress resistance in a pathogenic yeast
Source: PLoS Pathog. 2023 Oct 23;19(10):e1011748. doi: 10.1371/journal.ppat.1011748 (PMC10621968; doi:10.1371/journal.ppat.1011748)
Supplement: S2 Fig — ASR experiment was performed as in Fig 1, with the exception of using different primary stresses as indicated on the top: Mock—rich SC medium; HS–Heat shock at 43C; -Glu—0.05% glucose (as opposed to 2% in SC); -Pi—no phosphate SC medium. Images were taken 14 hrs and 21 hrs post spotting for C. glabrata and S. cerevisiae respectively. (PDF) [file ppat.1011748.s002.pdf]

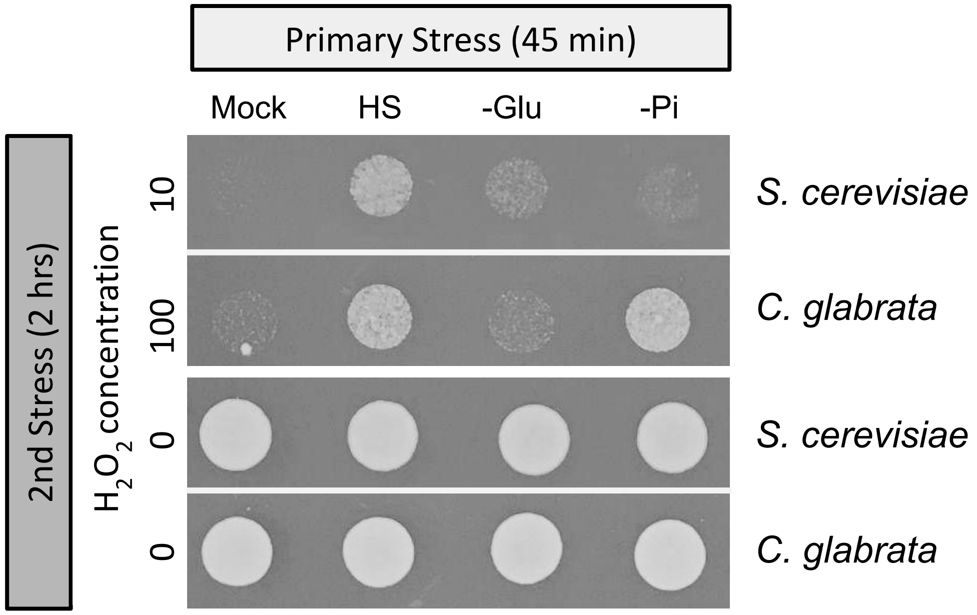

Supplementary Figure 2. Phosphate starvation-induced ASR for H_2O_2 diverge between species while other primary stresses show similar effects. ASR experiment was performed as in Figure 1, with the exception of using different primary stresses as indicated on the top: Mock - rich SC medium; HS - Heat shock at 43C; -Glu - 0.05% glucose (as opposed to 2% in SC); -Pi - no phosphate SC medium. Images were taken 14 hrs and 21 hrs post spotting for *C. glabrata* and *S. cerevisiae* respectively.
