# Supplementary material for: Divergence of TORC1-mediated stress response leads to novel acquired stress resistance in a pathogenic yeast
Source: PLoS Pathog. 2023 Oct 23;19(10):e1011748. doi: 10.1371/journal.ppat.1011748 (PMC10621968; doi:10.1371/journal.ppat.1011748)
Supplement: S3 Fig — We tested the ability of phosphate starvation to provide acquired resistance for two additional ROS, i.e., tert-butyl hydroperoxide and menadione sodium bisulfate (MSB), and compared them to H2O2, each at the indicated concentration. No ASR effect was observed for tBOOH (mean ASR-score = 0.71, 95% CI [0.49, 0.88], P = 1). There is moderate ASR for MSB (mean ASR-score = 2.41, 95% CI [1.79, 3.00], raw P = 0.016). ASR for H2O2 is the strongest (mean ASR-score = 16.4, 95% CI [7.7, 25.3], raw P = 0.031). (PDF) [file ppat.1011748.s003.pdf]

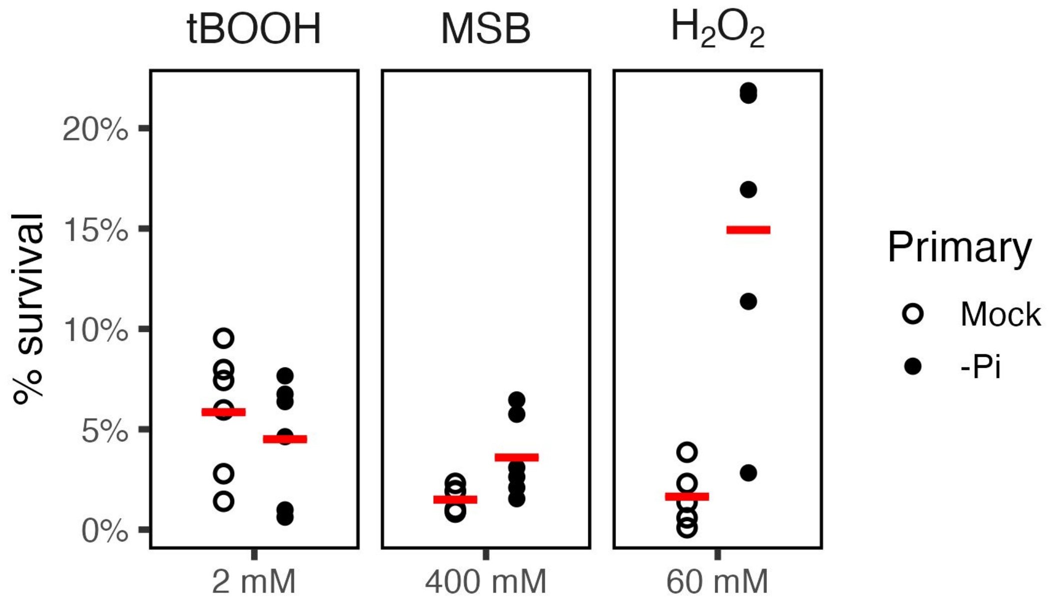

Supplementary Figure 3. Phosphate starvation's ASR effect depends on the type of ROS. We tested the ability of phosphate starvation to provide acquired resistance for two additional ROS, i.e., tert-butyl hydroperoxide and menadione sodium bisulfate (MSB), and compared them to H_2O_2, each at the indicated concentration. No ASR effect was observed for tBOOH (mean ASR-score = 0.71, 95% CI [0.49, 0.88], P = 1). There is moderate ASR for MSB (mean ASR-score = 2.41, 95% CI [1.79, 3.00], raw P = 0.016). ASR for H_2O_2 is the strongest (mean ASR-score = 16.4, 95% CI [7.7, 25.3], raw P = 0.031).
