# Supplementary material for: Divergence of TORC1-mediated stress response leads to novel acquired stress resistance in a pathogenic yeast
Source: PLoS Pathog. 2023 Oct 23;19(10):e1011748. doi: 10.1371/journal.ppat.1011748 (PMC10621968; doi:10.1371/journal.ppat.1011748)
Supplement: S4 Fig — Putting either CTA1-GFP (A) or the untagged CTA1 (B) back to the endogenous locus in the cta1Δ background restored the resistance to H2O2 compared with the wild type strain. Each strain was treated at the indicated concentrations of H2O2 for 2 hours, then spotted onto YPD plates and incubated at 30°C for 48 hours (A) and 20hrs (B). (C) ASR in the wild type, CTA1 complement (cta1::CTA1) and cta1Δ strains. The experiment was conducted similarly as in Fig 3 for wild type and cta1Δ strains. Concentrations of H2O2 were calibrated to achieve a similar basal survival rates (open circles, Kruskal-Wallis rank sum test for differences among the three groups P = 0.38). ASR-scores for the three strains are (with 95% CI and Wilcoxon signed-rank test P-values in the parenthesis): wild type 23.9 ([14.9, 36.5], P = 0.048); cta1::CTA1 15.4 ([9.5, 21.3], P = 0.048); cta1Δ 5.1 ([2.0, 10.6], P = 0.093). The difference in ASR-score is significant between cta1Δ and wild type, but not between cta1::CTA1 and wild type (Mann-Whitney U test P = 0.034 and 0.48, respectively, Bonferroni-corrected). (PDF) [file ppat.1011748.s004.pdf]

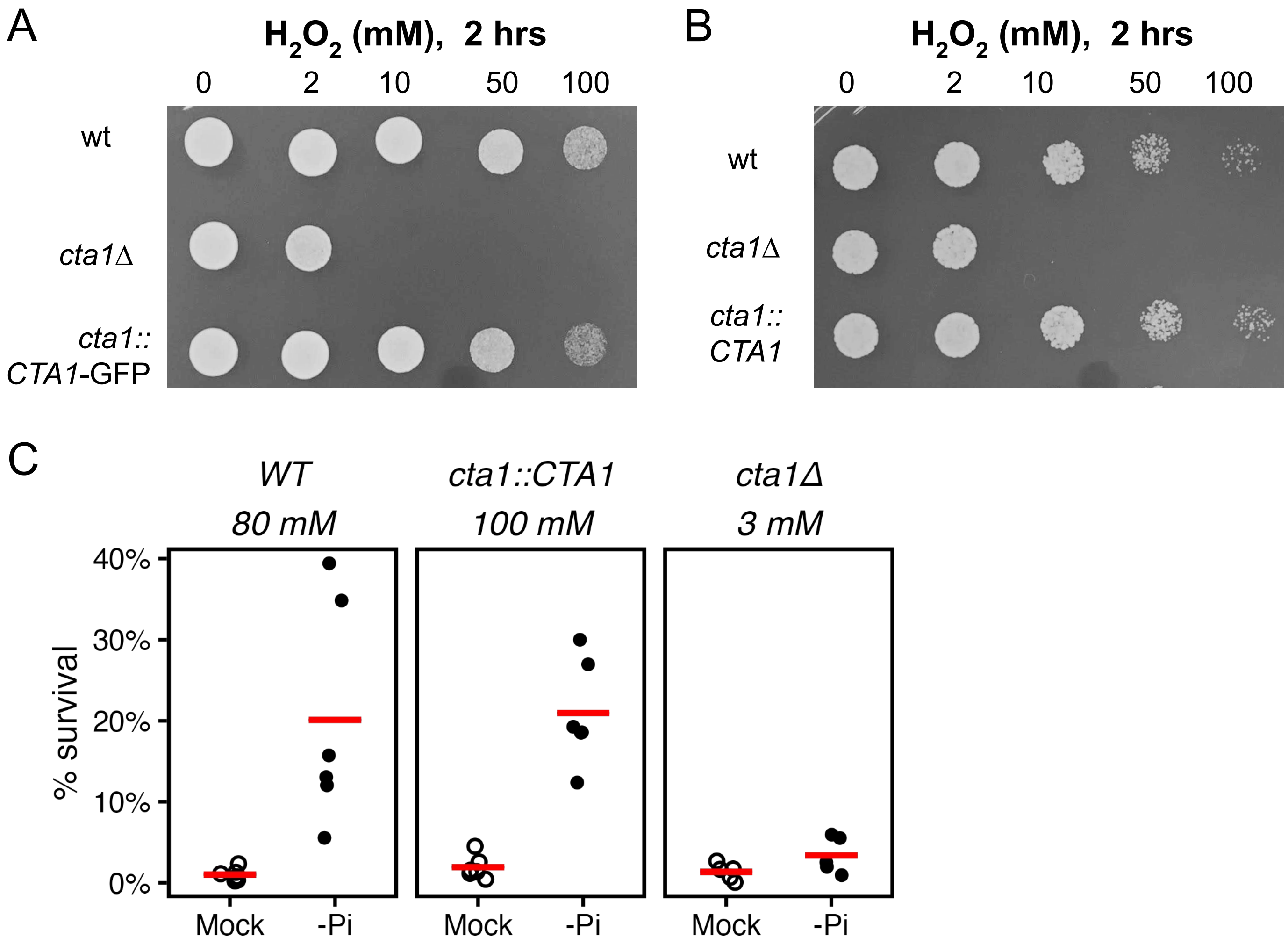

Supplementary Figure 4. ***CTA1*** **complement strain rescues basal H_2O_2 survival and ASR defects in** ***cta1*****Δ.** Putting either *CTA1*-GFP (A) or the untagged *CTA1* (B) back to the endogenous locus in the *cta1*Δ background restored the resistance to H_2O_2 compared with the wild type strain. Each strain was treated at the indicated concentrations of H_2O_2 for 2 hours, then spotted onto YPD plates and incubated at 30°C for 48 hours (A) and 20hrs (B). (C) ASR in the wild type, *CTA1* complement (*cta1::CTA1*) and *cta1*Δ strains. The experiment was conducted similarly as in Fig. 3 for wild type and *cta1*Δ strains. Concentrations of H_2O_2 were calibrated to achieve a similar basal survival rates (open circles, Kruskal-Wallis rank sum test for differences among the three groups P = 0.38). ASR-scores for the three strains are (with 95% CI and Wilcoxon signed-rank test P-values in the parenthesis): wild type 23.9 ([14.9, 36.5], P=0.048); *cta1::CTA1* 15.4 ([9.5, 21.3], P=0.048); *cta1*Δ 5.1 ([2.0, 10.6], P=0.093). The difference in ASR-score is significant between *cta1*Δ and wild type, but not between *cta1::CTA1* and wild type (Mann-Whitney U test P = 0.034 and 0.48, respectively, Bonferroni-corrected).
