# Supplementary material for: Divergence of TORC1-mediated stress response leads to novel acquired stress resistance in a pathogenic yeast
Source: PLoS Pathog. 2023 Oct 23;19(10):e1011748. doi: 10.1371/journal.ppat.1011748 (PMC10621968; doi:10.1371/journal.ppat.1011748)
Supplement: S5 Fig — To test if pre-inducing CTA1 is sufficient to provide ASR for H2O2, we replaced the endogenous CTA1 promoter with the promoter of the C. glabrata MET3 gene. When grown in SC medium lacking methionine and cysteine (-MC), CTA1 was induced to a comparable level as in the endogenous CTA1pr-CTA1 strain under phosphate starvation at 45 minutes (A). Dots represent the mean of at least 3 biological replicates, and the error bars the 95% confidence interval by bootstrapping. The line is the LOESS fit to the data. The endogenous CTA1pr-CTA1 has a basal expression level that is higher than the MET3pr-CTA1 (0 min). We also confirmed that the -MC media itself did not provide ASR in the wild type C. glabrata cells (B, top). Note that in this set of ASR experiments, all SC medium containing H2O2 also lacked methionine and cysteine (C). This allows the MET3pr-CTA1 to be induced during the secondary stress, mimicking what the wild type strain experiences during the H2O2 stress. Using this strain, we found that inducing CTA1 during the primary stress significantly enhanced the survival of C. glabrata cells during the secondary oxidative stress, i.e., providing ASR (B, bottom two rows, -MC vs Mock). (PDF) [file ppat.1011748.s005.pdf]

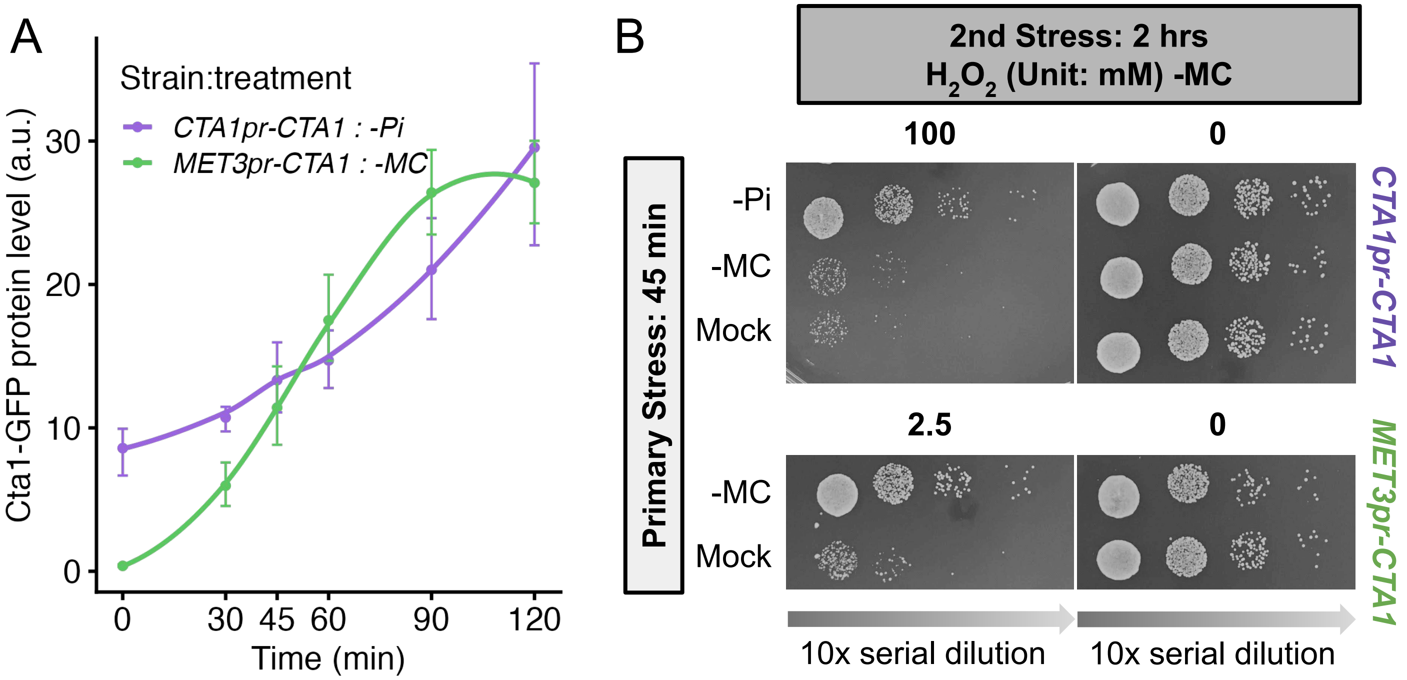

C

Primary stress (45 min)	Secondary Stress (2 hrs)
-Pi : SC w/o Pi	Oxidative: H_2O_2 added to -MC medium
-MC: SC w/o methionine and cysteine	Mock: SC medium
Mock: SC medium	

Supplementary Figure 5. Induction of *CTA1* provides acquired resistance to H_2O_2 in *C. glabrata*. To test if pre-inducing *CTA1* is sufficient to provide ASR for H_2O_2, we replaced the endogenous *CTA1* promoter with the promoter of the *C. glabrata MET3* gene. When grown in SC medium lacking methionine and cysteine (-MC), *CTA1* was induced to a comparable level as in the endogenous *CTA1pr-CTA1* strain under phosphate starvation at 45 minutes (A). Dots represent the mean of at least 3 biological replicates, and the error bars the 95% confidence interval by bootstrapping. The line is the LOESS fit to the data. The endogenous *CTA1pr-CTA1* has a basal expression level that is higher than the *MET3pr-CTA1* (0 min). We also confirmed that the -MC media itself did not provide ASR in the wild type *C. glabrata* cells (B, top). Note that in this set of ASR experiments, all SC medium containing H_2O_2 also lacked methionine and cysteine (C). This allows the *MET3pr-CTA1* to be induced during the secondary stress, mimicking what the wild type strain experiences during the H_2O_2 stress. Using this strain, we found that inducing *CTA1* during the primary stress significantly enhanced the survival of *C. glabrata* cells during the secondary oxidative stress, i.e., providing ASR (B, bottom two rows, -MC vs Mock).
