# Supplementary material for: Divergence of TORC1-mediated stress response leads to novel acquired stress resistance in a pathogenic yeast
Source: PLoS Pathog. 2023 Oct 23;19(10):e1011748. doi: 10.1371/journal.ppat.1011748 (PMC10621968; doi:10.1371/journal.ppat.1011748)
Supplement: S6 Fig — (A) (Left) Deleting cta1 and the OSR TF, yap1, had no defect on survival under phosphate starvation, while deletion of pho4, which is responsible for the PHO response, showed severe growth defects. Mid-log phase cells of the indicated genotypes were spotted on no phosphate SC plates, incubated at 30°C for 48 hours. (Right) Same as (Left) but spotted onto SC plates with 7.5 mM Pi. All images are representatives of >3 biological replicates. (B) CTA1’s importance for ASR is dependent on the primary stress type. ASR for wild type and cta1Δ were tested with various primary stresses, including phosphate starvation (-Pi), glucose starvation (-Glu, 0.02% glucose), mild H2O2 (1.5 mM H2O2). ASR experiment was performed as described in the text. (PDF) [file ppat.1011748.s006.pdf]

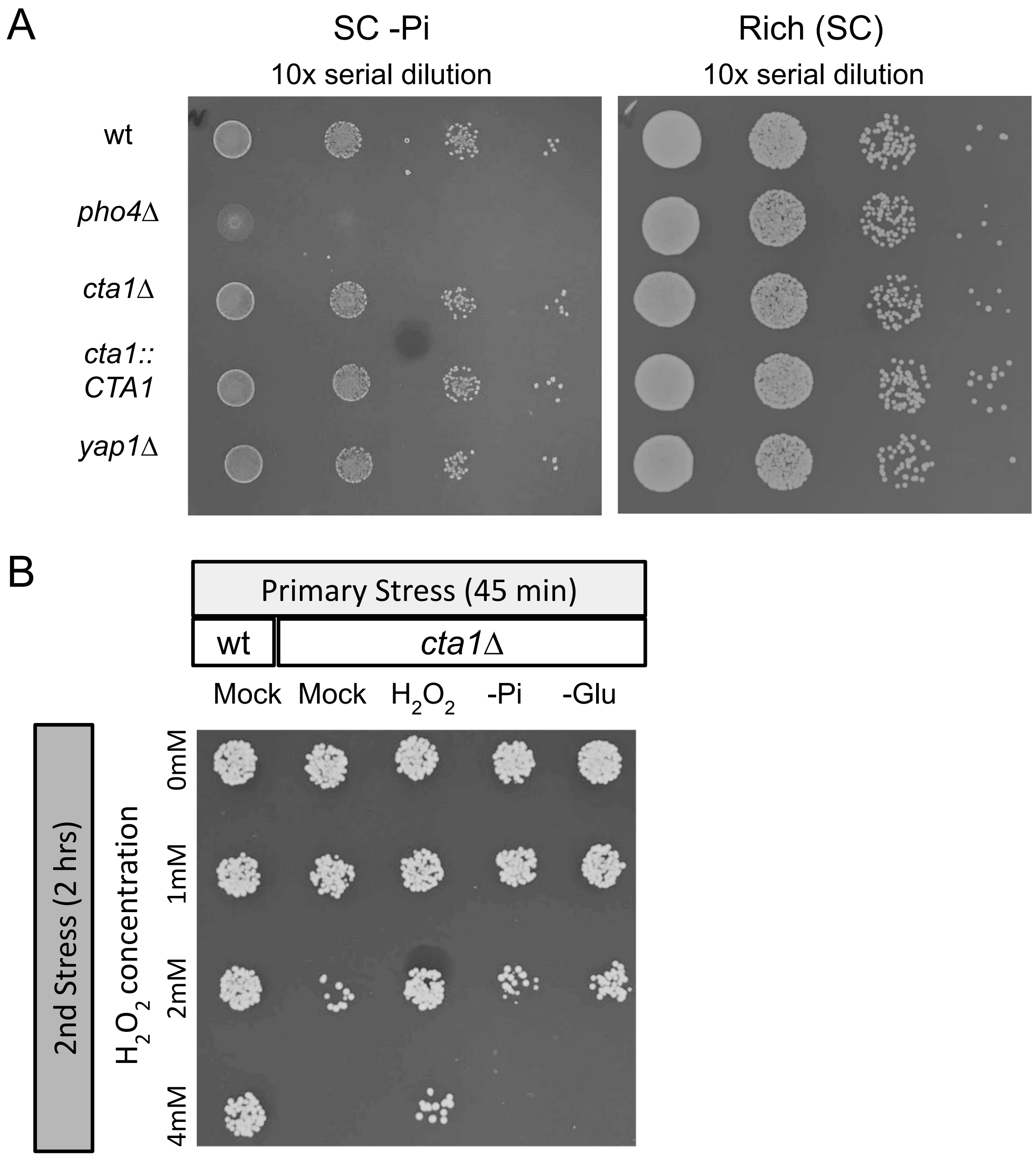

Supplementary Figure 6. Cta1 is not required for survival during the primary phosphate starvation, and is not as important for ASR with mild H_2O_2 used as the primary stress. (A) (Left) Deleting *cta1* and the OSR TF, *yap1*, had no defect on survival under phosphate starvation, while deletion of *pho4*, which is responsible for the PHO response, showed severe growth defects. Mid-log phase cells of the indicated genotypes were spotted on no phosphate SC plates, incubated at 30°C for 48 hours. (Right) Same as (Left) but spotted onto SC plates with 7.5 mM Pi. All images are representatives of >3 biological replicates. (B) *CTA1*'s importance for ASR is dependent on the primary stress type. ASR for wild type and *cta1*Δ were tested with various primary stresses, including phosphate starvation (-Pi), glucose starvation (-Glu, 0.02% glucose), mild H_2O_2 (1.5 mM H_2O_2). ASR experiment was performed as described in the text.
