# Supplementary material for: Divergence of TORC1-mediated stress response leads to novel acquired stress resistance in a pathogenic yeast
Source: PLoS Pathog. 2023 Oct 23;19(10):e1011748. doi: 10.1371/journal.ppat.1011748 (PMC10621968; doi:10.1371/journal.ppat.1011748)
Supplement: S7 Fig — Cta1-GFP induction under 2 mM H2O2 for 4 hours. Yap1, Skn7 play a critical role; Msn4 has a minor contribution while Msn2 is not important. Same features as in Fig 4A. (PDF) [file ppat.1011748.s007.pdf]

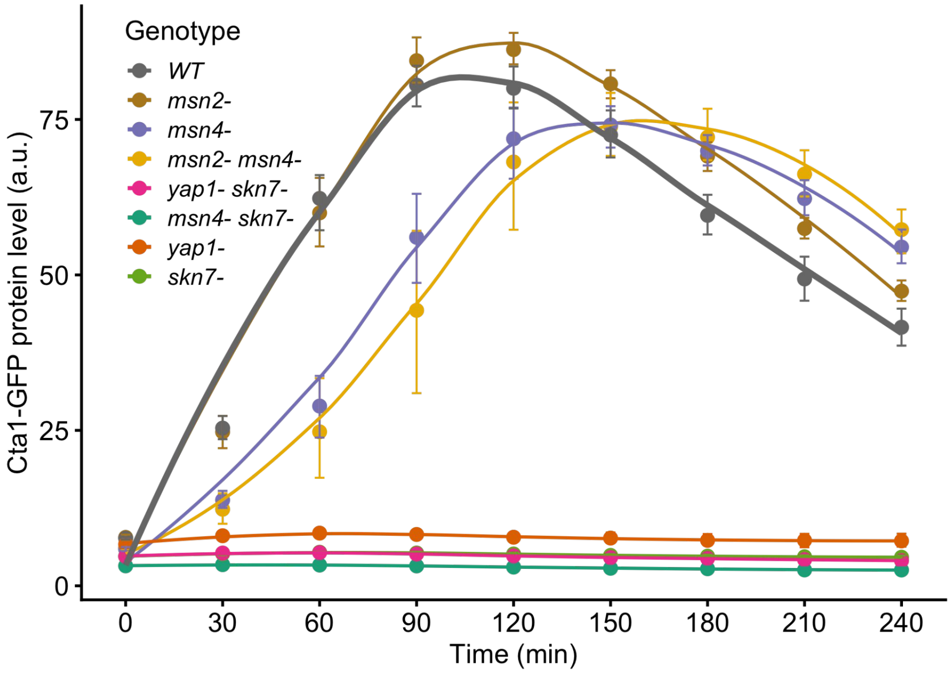

Supplementary Figure 7. TFΔ effects on Cta1-GFP induction under H_2O_2 stress. Cta1-GFP induction under 2 mM H_2O_2 for 4 hours. Yap1, Skn7 play a critical role; Msn4 has a minor contribution while Msn2 is not important. Same features as in Figure 4A.
