# Supplementary material for: Divergence of TORC1-mediated stress response leads to novel acquired stress resistance in a pathogenic yeast
Source: PLoS Pathog. 2023 Oct 23;19(10):e1011748. doi: 10.1371/journal.ppat.1011748 (PMC10621968; doi:10.1371/journal.ppat.1011748)
Supplement: S8 Fig — Cellular localization of CgMsn2 (A) and ScMsn2 (B) under phosphate starvation (-Pi) and no stress conditions. All treatments were for 45 minutes. From left to right: i. CgMsn2-yeGFP and ScMsn2-mCherry (pseudo color); ii. nucleus staining with DAPI in C. glabrata or Nh6a-iRFP in S. cerevisiae; iii. merge of i and ii; iv. bright field; v. percentage of cells with nuclear-localized CgMsn2 or ScMsn2 under each condition (n = number of cells quantified). Scale bars all represent 5 μm in length. DAPI stain more than the nucleus in live C. glabrata cells as observed by others [42]. (PDF) [file ppat.1011748.s008.pdf]

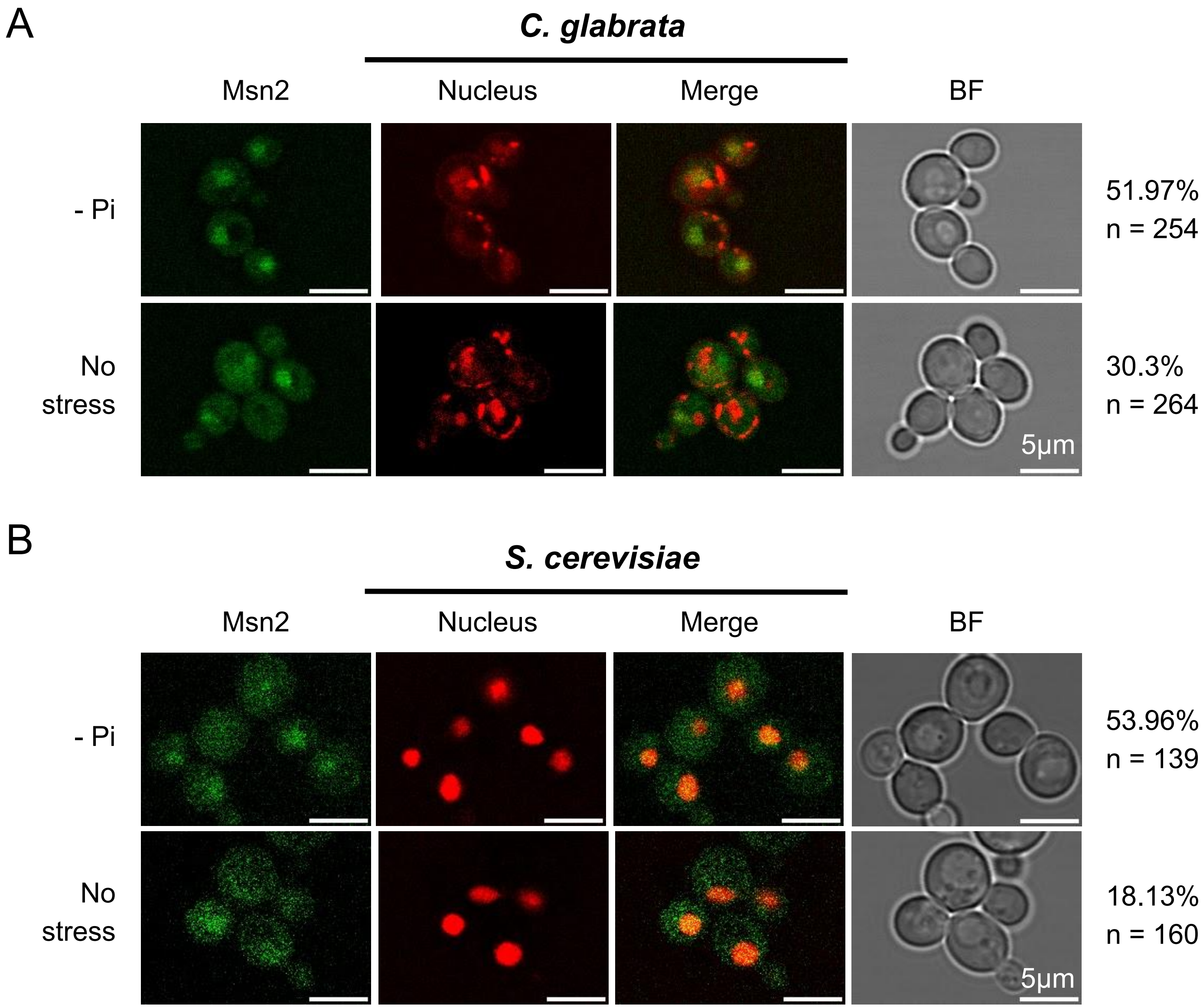

Supplementary Figure 8. *C. glabrata* Msn2 (CgMsn2) and *S. cerevisiae* Msn2 (ScMsn2) translocate into the nucleus upon phosphate starvation. Cellular localization of CgMsn2 (A) and ScMsn2 (B) under phosphate starvation (-Pi) and no stress conditions. All treatments were for 45 minutes. From left to right: i. CgMsn2-yeGFP and ScMsn2-mCherry (pseudo color); ii. nucleus staining with DAPI in *C. glabrata* or Nh6a-iRFP in *S. cerevisiae*; iii. merge of i and ii; iv. bright field; v. percentage of cells with nuclear-localized CgMsn2 or ScMsn2 under each condition (n=number of cells quantified). Scale bars all represent 5 μm in length. DAPI stain more than the nucleus in live *C. glabrata* cells as observed by others (Roetzer *et al.* 2008).
