# Supplementary material for: Divergence of TORC1-mediated stress response leads to novel acquired stress resistance in a pathogenic yeast
Source: PLoS Pathog. 2023 Oct 23;19(10):e1011748. doi: 10.1371/journal.ppat.1011748 (PMC10621968; doi:10.1371/journal.ppat.1011748)
Supplement: S9 Fig — We restored RIM15 at the endogenous locus on the rim15Δ background and compared its ASR effect to that in the wild type and rim15Δ strains. Concentrations of H2O2 were calibrated to achieve a similar basal survival rates (open circles, Kruskal-Wallis rank sum test among the three groups P = 0.27). ASR-scores for the three strains are (95% CI and Wilcoxon signed-rank test P-values in the parenthesis): wild type 9.5 ([5.3, 16.9], P = 0.048); rim15::RIM15 11.6 ([8.6, 15.2], P = 0.048); rim15Δ 5.3 ([3.4, 7.5], P = 0.048). Based on Mann-Whitney U test, the ASR-score is not significantly different between either rim15Δ and wild type, or rim15::RIM15 and wild type (Holm-Bonferroni-corrected P-values = 0.6 and 0.3, respectively). rim15Δ has relatively weak effects on ASR (Fig 6D) and the wild type’s survival rates with primary stress has relatively large variance in this experiment. Both could result in the lack of statistical significance in the comparison between rim15Δ and wild type. Instead, the difference in ASR-score between rim15Δ and rim15::RIM15 has a P-value of 0.045. Combined, we conclude that rim15Δ reduces ASR and the complement strain rescues the defect. (PDF) [file ppat.1011748.s009.pdf]

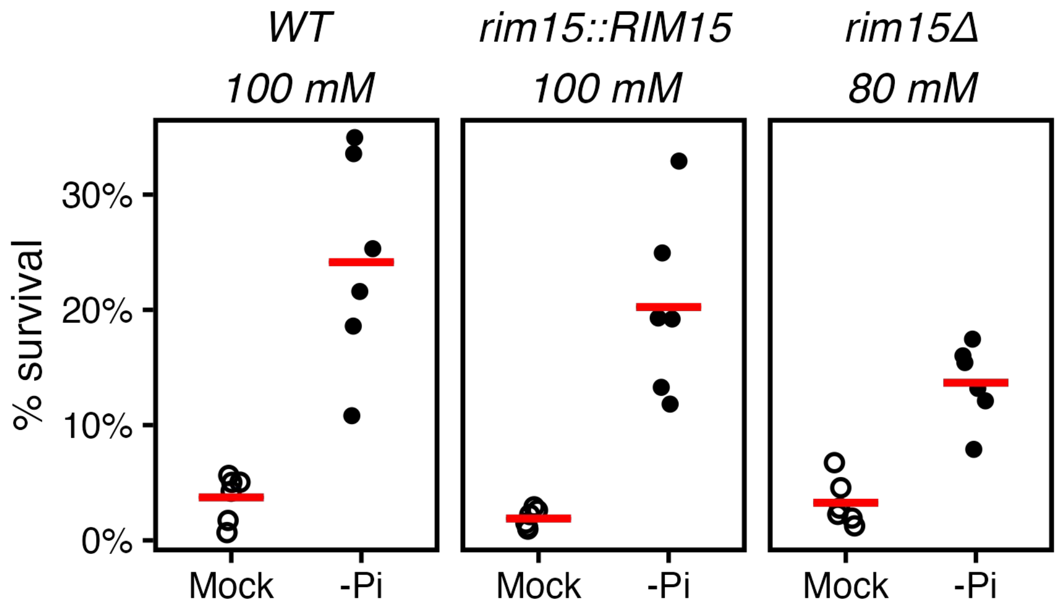

Supplementary Figure 9. *RIM15* complement strain rescues *rim15*Δ ASR defects. We restored *RIM15* at the endogenous locus on the *rim15*Δ background and compared its ASR effect to that in the wild type and *rim15*Δ strains. Concentrations of H_2O_2 were calibrated to achieve a similar basal survival rates (open circles, Kruskal-Wallis rank sum test among the three groups P = 0.27). ASR-scores for the three strains are (95% CI and Wilcoxon signed-rank test P-values in the parenthesis): wild type 9.5 ([5.3, 16.9], P=0.048); *rim15::RIM15* 11.6 ([8.6, 15.2], P=0.048); *rim15*Δ 5.3 ([3.4, 7.5], P=0.048). Based on Mann-Whitney U test, the ASR-score is not significantly different between either *rim15*Δ and wild type, or *rim15::RIM15* and wild type (Holm-Bonferroni-corrected P-values = 0.6 and 0.3, respectively). *rim15*Δ has relatively weak effects on ASR (Fig. 6D) and the wild type's survival rates with primary stress has relatively large variance in this experiment. Both could result in the lack of statistical significance in the comparison between *rim15*Δ and wild type. Instead, the difference in ASR-score between *rim15*Δ and *rim15::RIM15* has a P-value of 0.045. Combined, we conclude that *rim15*Δ reduces ASR and the complement strain rescues the defect.
