# Supplementary material for: Divergence of TORC1-mediated stress response leads to novel acquired stress resistance in a pathogenic yeast
Source: PLoS Pathog. 2023 Oct 23;19(10):e1011748. doi: 10.1371/journal.ppat.1011748 (PMC10621968; doi:10.1371/journal.ppat.1011748)
Supplement: S10 Fig — (A) Western blot for P-Rps6 (top) and total Rps6 (bottom) in log phase C. glabrata, S. cerevisiae, K. lactis and L. walti cells, grown in rich, nitrogen starvation (-N) and phosphate starvation (-Pi) media for 1 hr at 30°C. This blot is representative of three biological replicates. The subset of the image including S. cerevisiae and C. glabrata was already shown in Fig 7A. The cladogram on the top depicts the phylogeny between the yeasts; the arrow on the top blot indicates the band for the P-Rps6; the two dotted lines in the bottom blot indicate the bands for total Rps6 used for quantitative analysis. (B) Quantification of the ratio of P-Rps6 to total Rps6 based on three replicates. While the trend was obvious, the small sample size (3) limited the power of a paired Student’s t-test used to compare the starvation conditions to the rich condition in each species, which resulted in P-values that were not significant at a 0.05 level after Bonferroni correction. (PDF) [file ppat.1011748.s010.pdf]

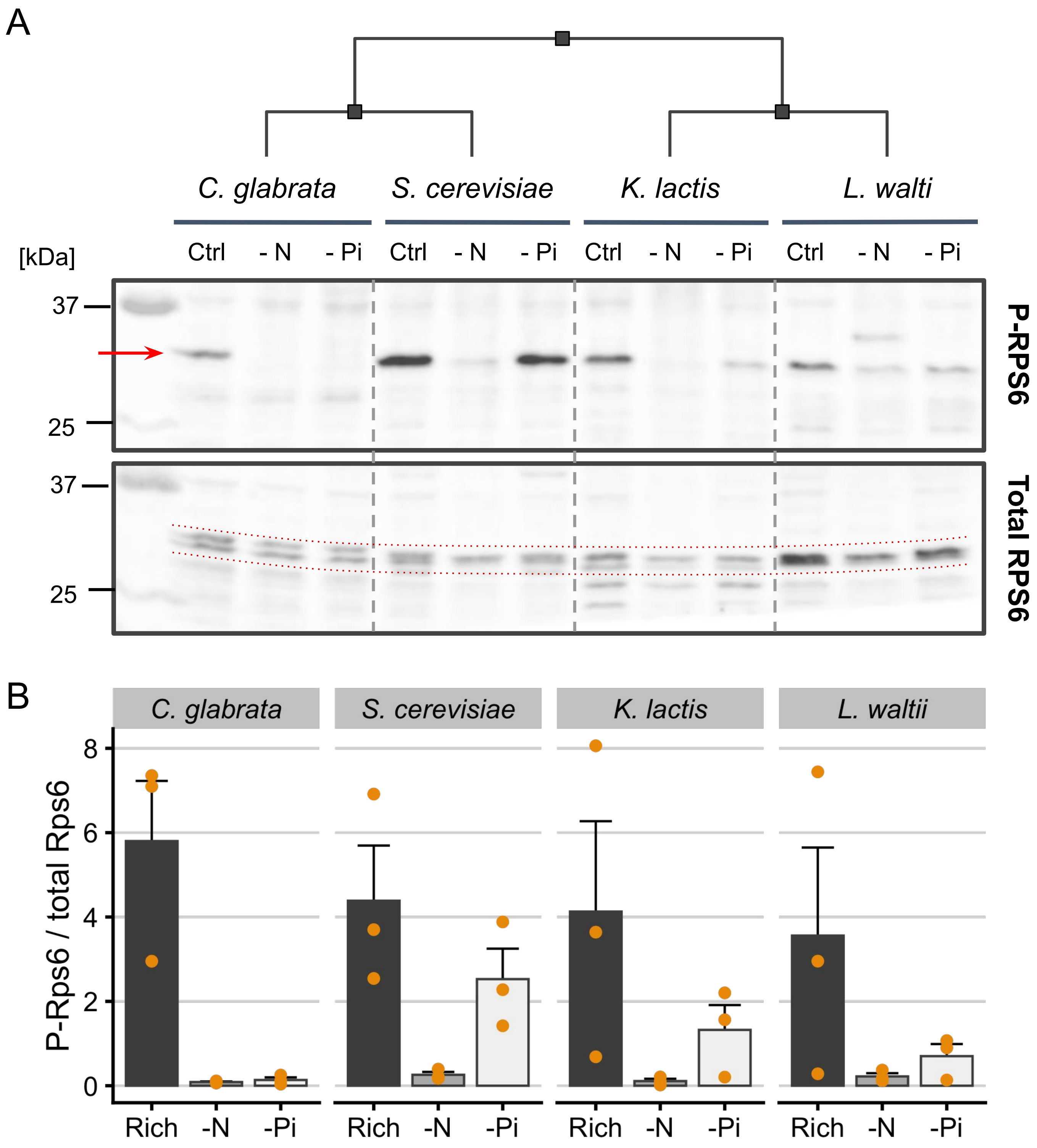

Supplementary Figure 10. TORC1 inhibition by phosphate and nitrogen starvation in diverse yeasts. (A) Western blot for P-Rps6 (top) and total Rps6 (bottom) in log phase *C. glabrata, S. cerevisiae, K. lactis* and *L. walti* cells, grown in rich, nitrogen starvation (-N) and phosphate starvation (-Pi) media for 1 hr at 30 ° C. This blot is representative of three biological replicates. The subset of the image including *S. cerevisiae* and *C. glabrata* was already shown in Fig. 7A. The cladogram on the top depicts the phylogeny between the yeasts; the arrow on the top blot indicates the band for the P-Rps6; the two dotted lines in the bottom blot indicate the bands for total Rps6 used for quantitative analysis. (B) Quantification of the ratio of P-Rps6 to total Rps6 based on three replicates. While the trend was obvious, the small sample size (3) limited the power of a paired Student's t-test used to compare the starvation conditions to the rich condition in each species, which resulted in *P*-values that were not significant at a 0.05 level after Bonferroni correction.
