# Supplementary material for: Divergence of TORC1-mediated stress response leads to novel acquired stress resistance in a pathogenic yeast
Source: PLoS Pathog. 2023 Oct 23;19(10):e1011748. doi: 10.1371/journal.ppat.1011748 (PMC10621968; doi:10.1371/journal.ppat.1011748)
Supplement: S11 Fig — (A) Schematic alignment of Sch9 protein in S. cerevisiae and C. glabrata. The three TORC1 targeted S/T sites in ScSch9, based on Urban et al. 2007, and the corresponding sites in the orthologous CgSch9 based on the alignment were labeled with a triangle and the mutations were labeled on the top. The region containing the three S/T sites were shown below as a pairwise sequence alignment below. The S/T sites were shown in red bold fonts. For the second site, S758 (in ScSch9), the corresponding site in CgSch9 are flanked by two serines, making it difficult to determine whether and which serine may be the authentic phosphorylation site. We therefore decided to make three mutations to turn the site into the same sequence as in the ScSCH9-3E mutant. (B) To replace the endogenous SCH9 gene in C. glabrata with the mutant alleles, we added an antibiotic NAT marker at the end of either the wild type CgSCH9 gene or the CgSCH9-3E allele and performed allele swaps. The first construct provides a control for the effect of disrupting the 3’ UTR by adding the NAT marker. (PDF) [file ppat.1011748.s011.pdf]

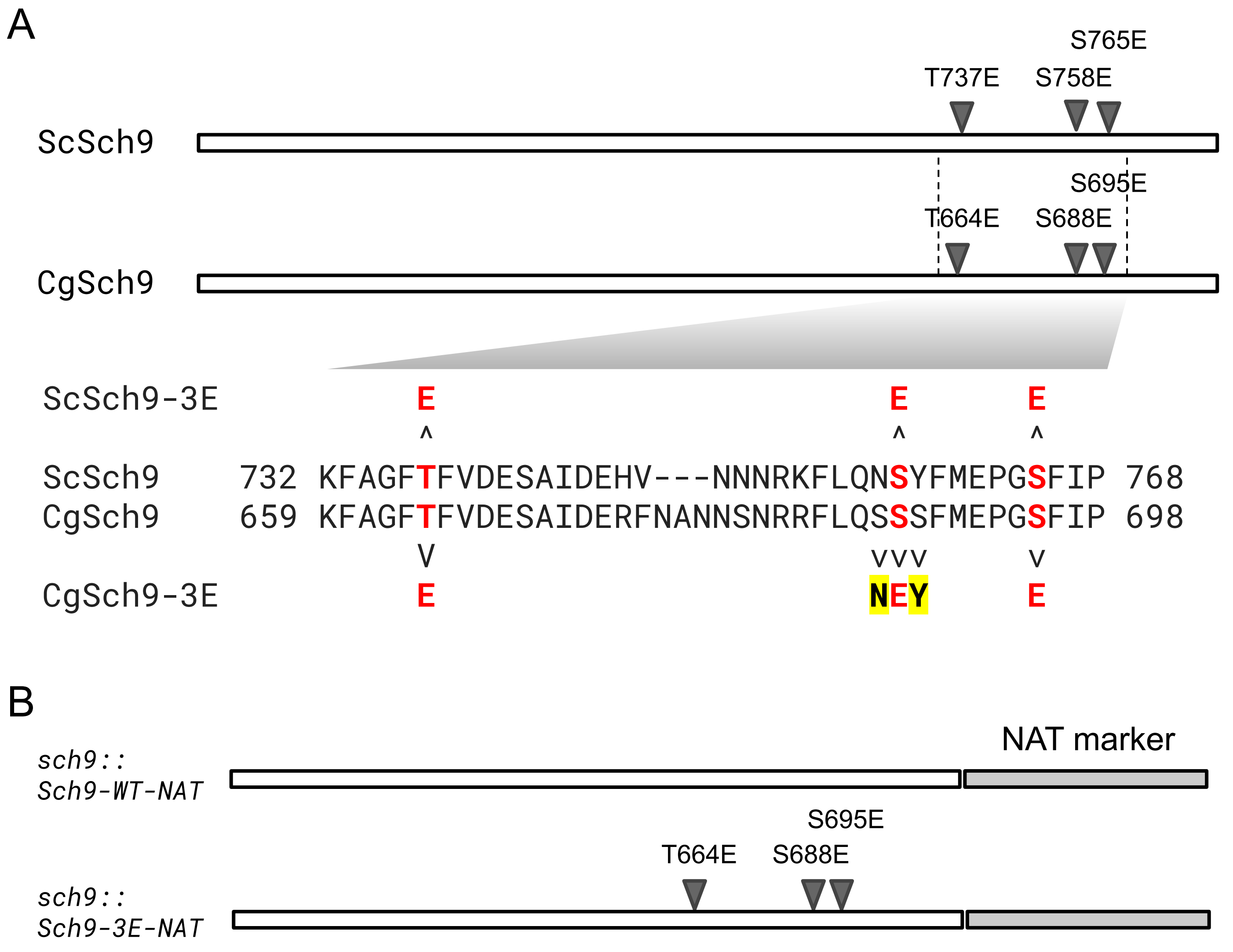

Supplementary Figure 11. Sch9-3E phosphomimetic mutant in *C. glabrata*. (A) Schematic alignment of Sch9 protein in *S. cerevisiae* and *C. glabrata*. The three TORC1 targeted S/T sites in ScSch9, based on Urban *et al.* 2007, and the corresponding sites in the orthologous CgSch9 based on the alignment were labeled with a triangle and the mutations were labeled on the top. The region containing the three S/T sites were shown below as a pairwise sequence alignment below. The S/T sites were shown in red bold fonts. For the second site, S758 (in ScSch9), the corresponding site in CgSch9 are flanked by two serines, making it difficult to determine whether and which serine may be the authentic phosphorylation site. We therefore decided to make three mutations to turn the site into the same sequence as in the ScSCH9-3E mutant. (B) To replace the endogenous *SCH9* gene in *C. glabrata* with the mutant alleles, we added an antibiotic NAT marker at the end of either the wild type *CgSCH9* gene or the *CgSCH9-3E* allele and performed allele swaps. The first construct provides a control for the effect of disrupting the 3' UTR by adding the NAT marker.
