# Supplementary material for: Divergence of TORC1-mediated stress response leads to novel acquired stress resistance in a pathogenic yeast
Source: PLoS Pathog. 2023 Oct 23;19(10):e1011748. doi: 10.1371/journal.ppat.1011748 (PMC10621968; doi:10.1371/journal.ppat.1011748)
Supplement: S12 Fig — ScDAL80pr-GFP expression levels were monitored via flow cytometry over a time course of 4 hours during nitrogen (-N) or phosphate starvation (-Pi) and rich medium conditions. Dots represent the mean of Median Fluorescent Intensity from > 3 biological replicates; the error bars represent the 95% CI based on 1000 bootstraps. The lines are LOESS fit to the means. The top row shows all three conditions while the bottom row shows the same data without -N to better visualize the induction under -Pi. (PDF) [file ppat.1011748.s012.pdf]

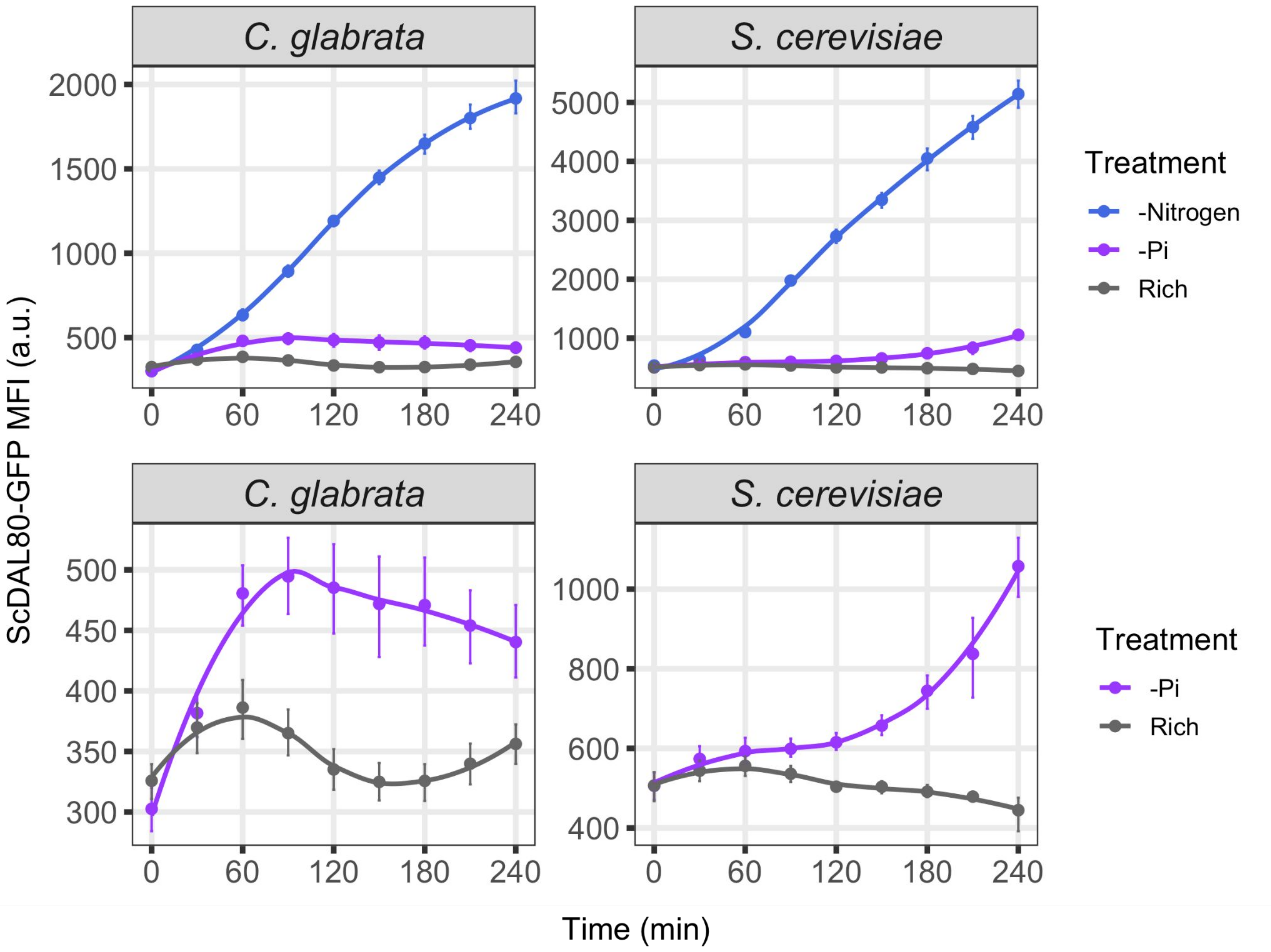

Supplementary Figure 12. *DAL80* induction kinetics under nitrogen and phosphate starvation in *S. cerevisiae* and *C. glabrata*. *ScDAL80pr*-GFP expression levels were monitored via flow cytometry over a time course of 4 hours during nitrogen (-N) or phosphate starvation (-Pi) and rich medium conditions. Dots represent the mean of Median Fluorescent Intensity from > 3 biological replicates; the error bars represent the 95% CI based on 1000 bootstraps. The lines are LOESS fit to the means. The top row shows all three conditions while the bottom row shows the same data without -N to better visualize the induction under -Pi.
