# Supplementary material for: Divergence of TORC1-mediated stress response leads to novel acquired stress resistance in a pathogenic yeast
Source: PLoS Pathog. 2023 Oct 23;19(10):e1011748. doi: 10.1371/journal.ppat.1011748 (PMC10621968; doi:10.1371/journal.ppat.1011748)
Supplement: S13 Fig — Shown are the phosphate homeostasis genes induced during phosphate starvation in C. glabrata. Gene list is from [25]. PolII ChIP-seq time course data are from [31], sampled at 0.5h, 2h, 4h, 6h and 8h post infection. For each gene, the ChIP-seq RPKM values were divided by the mean such that the normalized values represent fold changes over the mean for that gene. Systematic gene IDs were labeled on the top of each panel. Gene names were shown inside the panel (names in parentheses were based on S. cerevisiae homologs). Colors represented gene functional categories as explained below. (PDF) [file ppat.1011748.s013.pdf]

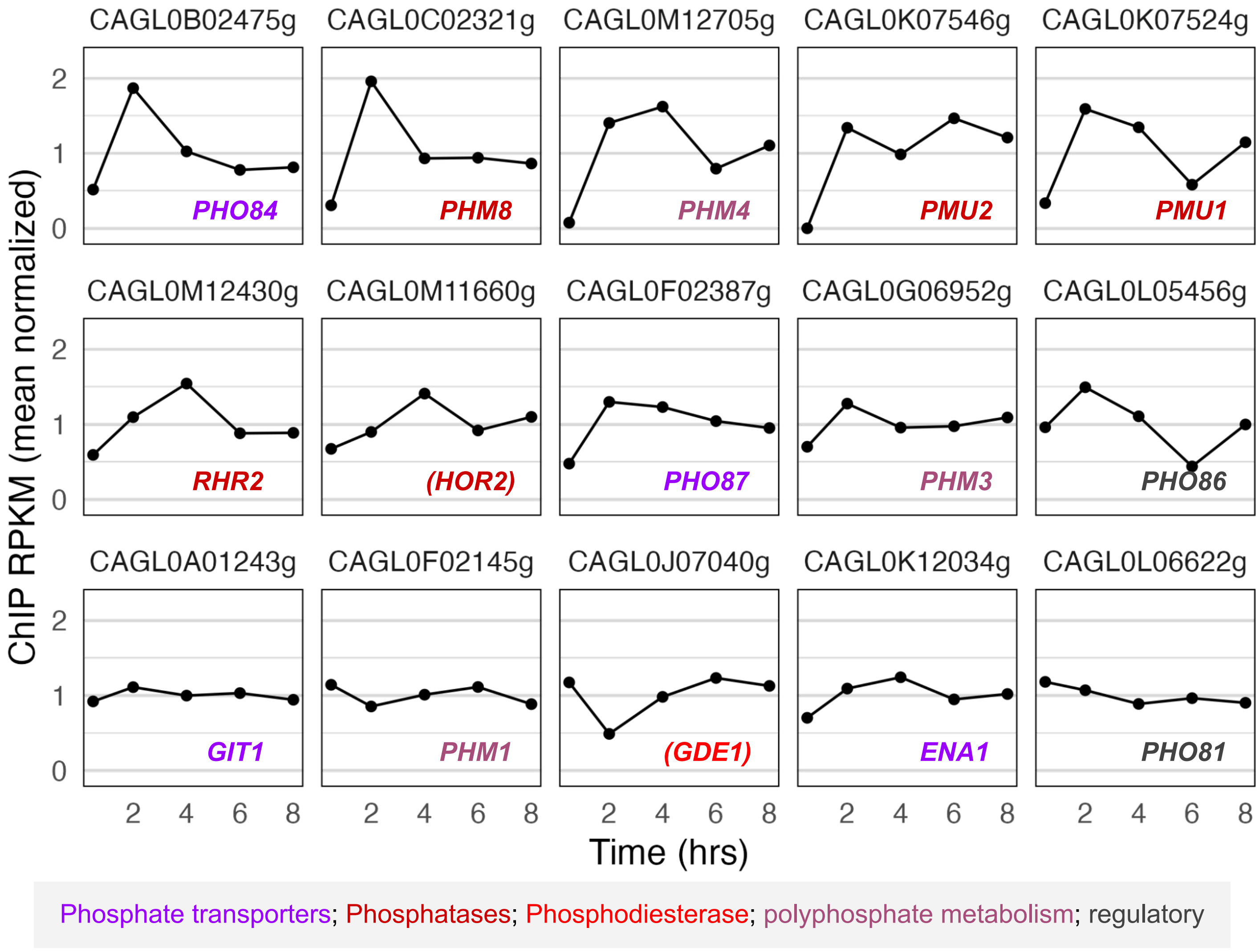

Supplementary Figure 13. Phosphate starvation response is partially induced in *C. glabrata* after the yeast is engulfed by human macrophages. Shown are the phosphate homeostasis genes induced during phosphate starvation in *C. glabrata*. Gene list is from (He *et al.* 2017). PolII ChIP-seq time course data are from (Rai *et al.* 2021), sampled at 0.5h, 2h, 4h, 6h and 8h post infection. For each gene, the ChIP-seq RPKM values were divided by the mean such that the normalized values represent fold changes over the mean for that gene. Systematic gene IDs were labeled on the top of each panel. Gene names were shown inside the panel (names in parentheses were based on *S. cerevisiae* homologs). Colors represented gene functional categories as explained below.
