# Supplementary material for: Divergence of TORC1-mediated stress response leads to novel acquired stress resistance in a pathogenic yeast
Source: PLoS Pathog. 2023 Oct 23;19(10):e1011748. doi: 10.1371/journal.ppat.1011748 (PMC10621968; doi:10.1371/journal.ppat.1011748)
Supplement: S14 Fig — Data and plots are similar to Fig 2C–2F. Gene sets were based on [31] (S2 and S3 Figs for Fig 1 therein). Gene names were based on S. cerevisiae. An asterisk meant the gene was significantly induced in the species at an FDR of 0.05. (PDF) [file ppat.1011748.s014.pdf]

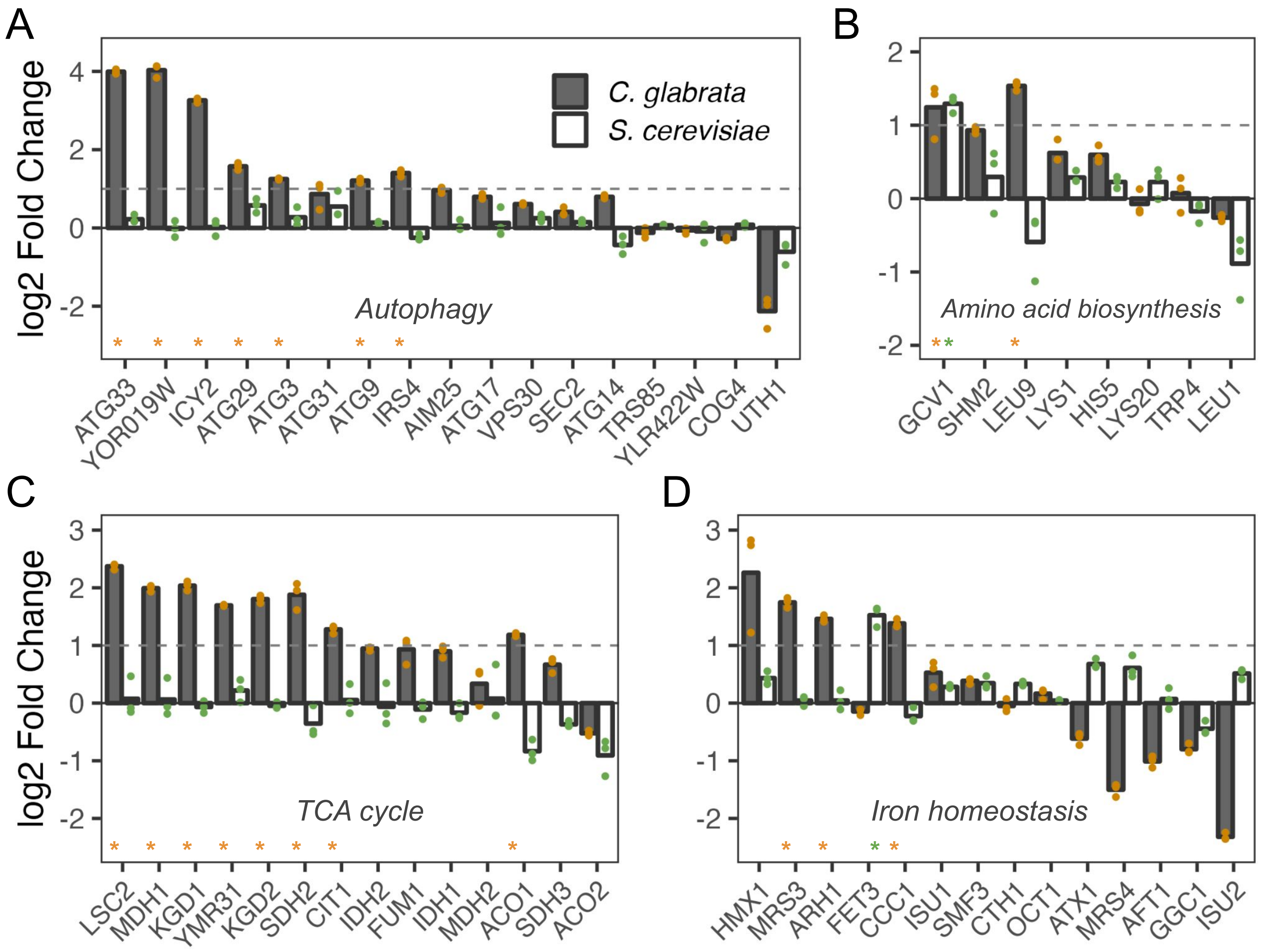

Supplementary figure 14. Genes induced after being engulfed by macrophages are also induced by a short-term phosphate starvation in *C. glabrata*. Data and plots are similar to Figure 2C-F. Gene sets were based on (Rai *et al.* 2021) supplementary figures 2 and 3 for figure 1. Gene names were based on *S. cerevisiae*. An asterisk meant the gene was significantly induced in the species at an FDR of 0.05.
