# Supplementary material for: Divergence of TORC1-mediated stress response leads to novel acquired stress resistance in a pathogenic yeast
Source: PLoS Pathog. 2023 Oct 23;19(10):e1011748. doi: 10.1371/journal.ppat.1011748 (PMC10621968; doi:10.1371/journal.ppat.1011748)
Supplement: S15 Fig — Cells were taken at 0, 20, 45, 75, 105 and 135 minutes during the time course and assayed for cell morphology by light microscopy (Materials and Methods). The black and dark red dots and lines represent two biological replicates. The dots are the percentage of unbudded cells from >100 total cells examined. The error bars are the 95% confidence interval for the binomial proportion based on normal approximation (Wald Interval). (PDF) [file ppat.1011748.s015.pdf]

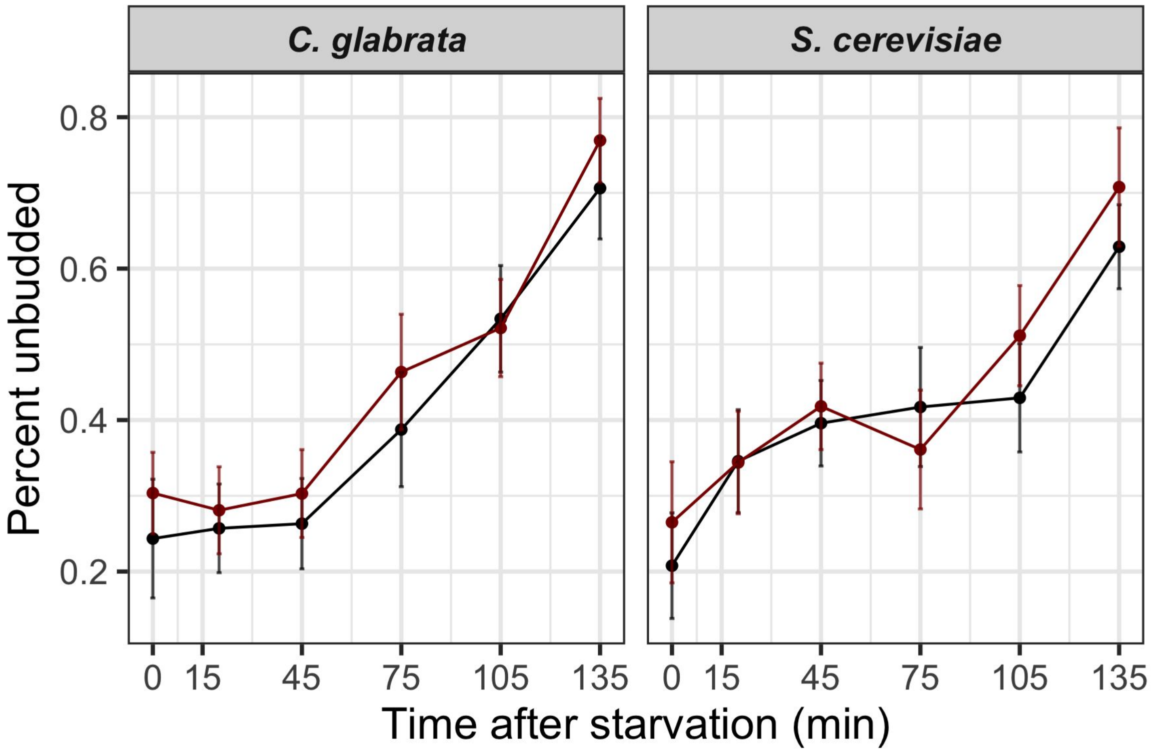

Supplementary Figure 15. Percent unbudded cells during phosphate starvation time course. Cells were taken at 0, 20, 45, 75, 105 and 135 minutes during the time course and assayed for cell morphology by light microscopy (Materials and Methods). The black and dark red dots and lines represent two biological replicates. The dots are the percentage of unbudded cells from >100 total cells examined. The error bars are the 95% confidence interval for the binomial proportion based on normal approximation (Wald Interval).
