# Supplementary material for: Divergence of TORC1-mediated stress response leads to novel acquired stress resistance in a pathogenic yeast
Source: PLoS Pathog. 2023 Oct 23;19(10):e1011748. doi: 10.1371/journal.ppat.1011748 (PMC10621968; doi:10.1371/journal.ppat.1011748)
Supplement: Attachment — Submitted filename: Response-to-reviewer-comments.pdf [file ppat.1011748.s019.pdf]

Dear Editor Dr. Olszewski,

We found the reviews constructive and extremely helpful. Thank you and all three anonymous reviewers for your input. Here, we provide detailed responses to each comment, starting from the main comments summarized in the editor's letter, reproduced below:

> *All three reviewers agree that the manuscript is important but requires further revisions. The most important set of comments pertains to validation of some of your data, including signal strength and imaging of the blots, absence of complement strain and determining the sufficiency of CTA1, and the use of only one type of oxidative stressor.*
> *Refining these issues will be important to solidify the important findings of this manuscript. Also, please make an effort to shorten the manuscript.*
> *This can be achieved by removing repetitive statements and focusing on the most important findings while limiting discussion outside of these major areas of focus.*

1. Signal strength and imaging of the blots.

> *From Reviewer #1: "Although the conclusions drawn from panel 7A remain convincing, the non-phospho RPS6 antibody does not give a clear signal, and thus it's unclear whether quantitation (Fig. 7B) is suitable. Were both major bands treated as RPS6 during quantification?"*

To clarify, the antibody for the second Western Blot recognizes both phosphorylated and non-phosphorylated Rps6, rather than just the non-phospho Rps6 (Fig. 7A, right). As the reviewer pointed out, there were two major bands adjacent to each other in the total Rps6 blot for *C. glabrata*, while a single or merged band existed for *S. cerevisiae*. All three bands were roughly the same size as the single band in the phosphorylated Rps6 blot, which was run in parallel with the total Rps6 blot (Fig. 7A). Therefore, we hypothesize that the two bands in *C. glabrata* both correspond to Rps6. Several reasons may account for the multiple bands, such as differential post-translational modification (PTM). Also, there are two *RPS6* genes in *C. glabrata* and *S. cerevisiae*, arising from the whole genome duplication (RPS6A and RPS6B). The two ohnologs have the same expected size of around 27 kDa, but may have differences in their PTM, which could also explain the banding pattern. Based on the above, we decided to quantify the total intensity of the single or double bands corresponding to the size of the single phosphorylated Rps6. Shown below are four versions of the same image. The top left is the original fluorescent Western image. The gray-scale version is shown below. On the right are the annotated versions

with boxes labeling the areas used for quantification.

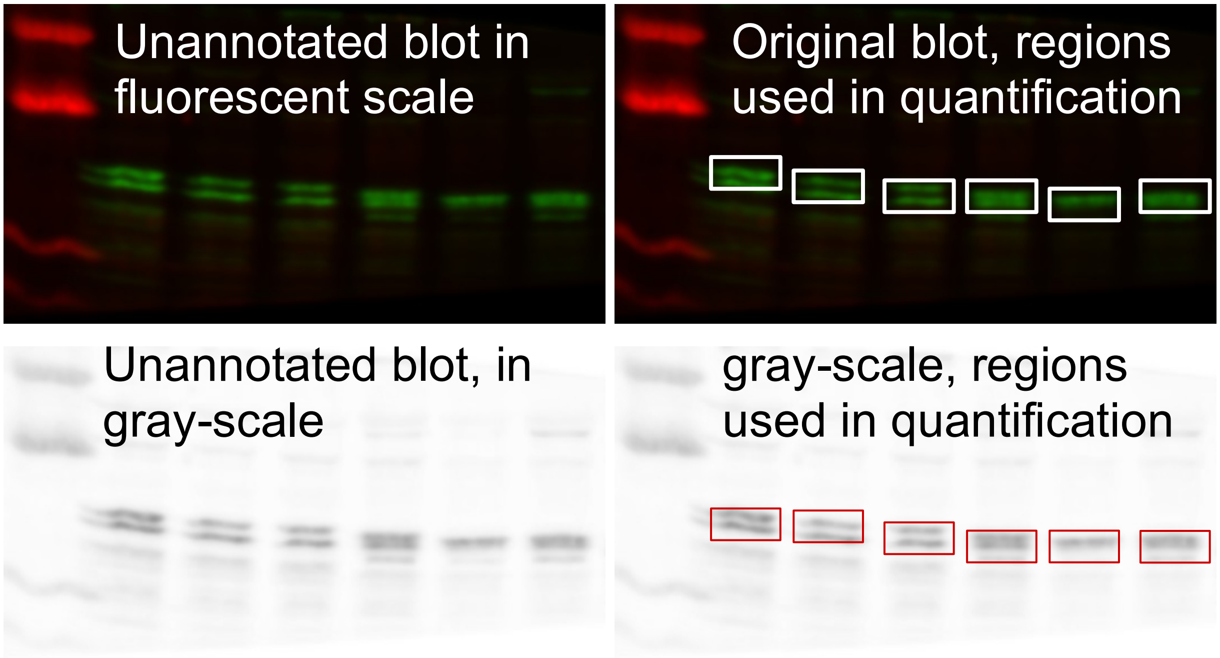

The intensity in each box was first background subtracted using the “top-bottom” setting in the ImageStudio software, then normalized by the corresponding total protein stain, according to the manufacturer’s protocol (LI-COR Revert™ 700 Total Protein Stain for Western Blot Normalization). The resulting value was used as the denominator to calculate P-Rps6 / Rps6, as shown in Fig. 7B and S10B.

To clarify how we quantified the blots, we annotated the total Rps6 blots in both Fig. 7A and Fig. S10A by adding two dotted lines to indicate the areas used for quantification. We also expanded the methods section for Western Blot to explain the procedures used. We hope this addresses the reviewer’s question and improves the reproducibility of our work.

2. Absence of complement strains

> *From Reviewer #2: “Finally, the reviewer’s concern is the lack of complemented strain. The inclusion of a few key complemented strains (such as CTA1) would strengthen the conclusions drawn from the knockout experiments.”*

We appreciate the reviewer’s comments and agree that complement strains will strengthen our conclusions. In the original submission, we measured ASR for three mutants: *cta1Δ*, *rim15Δ* and Sch9-3E. A complement strain for the last one was already included (sch9::SCH9-NAT, Fig. 7). In this revision, we constructed complement strains for the first two and compared them a new set of ASR experiments to both the deletion and the wild type. Both strains were constructed by replacing the URA3 cassette in the knock out strain with the original CDS sequence, restoring the locus to the wild type genotype. The strains were confirmed by PCR on both ends of the insertion and then subjected to quantitative ASR assay. We found that both complement strains rescued the ASR defects in the corresponding deletion mutants, confirming that the observed

defects were due to the intended deletion and not passenger mutations. We described the results in detail in the new Fig. S4 and S9.

3. Determine the sufficiency of *CTA1*

> *From Reviewer #2: "One concern regarding the study is the lack of clarity on whether the induction of CTA1 alone is sufficient to confer acquired stress resistance (ASR) to H2O2. While the study indicates that CgCTA1 induction is required for ASR during phosphate starvation, it does not directly address whether CTA1 induction alone is adequate to establish the resistance phenotype. Further investigations, such as genetic manipulation experiments, are needed to determine the sufficiency of CTA1 induction in conferring ASR for H2O2."*

We appreciate the reviewer's suggestion. To test if *CTA1* induction is sufficient to provide ASR for H_2O_2, we replaced the endogenous *CTA1* promoter with an inducible *MET3* promoter from *C. glabrata*. Using the endogenous C-terminal GFP tag, we confirmed that *CTA1* is induced in the induction medium (SC-Met-Cys), and that the level of induction is comparable to that after the same length of treatment under phosphate starvation (Fig. S5A). Using spotting assay, we found that pre-inducing *CTA1* is sufficient to provide ASR for the secondary H_2O_2 stress, while the same SC-Met-Cys medium doesn't provide ASR in a control strain where *CTA1* is driven by the endogenous promoter (Fig. S5). Therefore, we conclude that the induction of *CTA1* provides ASR for H_2O_2 in *C. glabrata*. It is worth noting that while *CTA1* clearly plays a critical role in the phosphate starvation-induced ASR for H_2O_2, our results, including this sufficiency test, do not rule out the contribution of other OSR genes induced under phosphate starvation.

4. The use of only one type of oxidative stressor

> *From Reviewer #3 "Screening on addtional oxidative stress chemicals post phophate starvation - is this limited to hydrogen peroxide only?" and "It would also be very interesting to see if the phosphate starvation also offered protection against other oxidative stressors such as tBOOH and how that effect the up/down regulation of Msn2/4 and Sch9."*

We appreciate the reviewer's suggestion. We agree that the suggested experiments will further elucidate the specificity of the ASR phenotype - whether it is general with respect to a class of secondary stressors or if it is very specific to a particular oxidant. We therefore tested tert-butyl hydroperoxide (tBOOH) as a model alkyl hydroperoxide, and menadione sodium bisulfite (MSB) as a model superoxide agent. The results were reported in Fig. S3 and discussed in the text. Briefly, we found that phosphate starvation provides no ASR for tBOOH while it has a moderate ASR effect for MSB in *C. glabrata*. The lack of ASR for tBOOH, while initially surprising, can be understood from its difference in chemical property and cellular target from H_2O_2. tBOOH is known to primarily cause lipid oxidation, disrupting the functions of the plasma membranes of yeast cells, and it activates a different TF than Yap1, named Cad1 (Iwai et al. 2010; Hajdu et al. 2021). There were also reports of tBOOH destroying catalases and other antioxidant activities, suggesting that the enzymes cells induce to decompose H_2O_2 may not be effective against this ROS (Pichorner et al. 1993; Ikeda et al. 2011). Combined, they may explain the different ASR

phenotypes between the two peroxide agents. For MSB, phosphate starvation does provide ASR but at a lower magnitude than H_2O_2. These observations show that ASR is highly stress-specific and not a general, cross-stress protection mechanism.

As for the reviewer's question on the up/down regulation of Msn2/4 and Sch9, we think there may be a misunderstanding of what we showed in our work. We showed that Msn4, but not Msn2, localizes into the nucleus, binds to *CTA1* promoter and induces its expression during phosphate starvation. We didn't show any results in the original manuscript with respect to their transcriptional or protein level changes during oxidative stress. We hope this clarifies the reviewer's question.

5. Shorten the manuscript

> *From Reviewer #3 "Overall this is a extremely well written manuscript, with all of the big hitting references on the topic cited, however it is in its current version, too long … The figures for the manuscript are of a very high standard but there is an overuse of schematics (F1, F2, F4, F6, F7, F8) a better way to present may be in one final figure describing the overall model presented herein."*

We thank the reviewer for the positive comment and appreciate the suggestion to streamline the manuscript. We made several changes in order to shorten the manuscript. For example, we moved the discussion of ASR genes having no or negative effects during the primary stress from Result to Discussion and merged it with existing texts. We also removed a supplementary figure on the effect of TFΔ on the basal expression levels of *CTA1*, as this result is not essential to our main conclusions. Additional changes can be seen in the tracked changes. However, due to the addition of figures and text in response to the reviewers' questions, the overall manuscript length wasn't significantly shorter (5207 to 5136 words, excluding bibliography and Materials and Methods).

We decided not to split the manuscript into two as Reviewer #3 suggested. Our reason is because the *S. cerevisiae* comparison serves a core purpose in our study, i.e., determine if ASR is evolvable and identify the main divergence points in the underlying network. To achieve this objective, species comparison is indispensable. We hope the reviewer would agree with us.

Response to additional reviewers' comments

Part I - Summary

Please use this section to discuss strengths/weaknesses of study, novelty/significance, general execution and scholarship.

From Reviewer #3: The figures for the manuscript are of a very high standard but there is an overuse of schematics (F1, F2, F4, F6, F7, F8) a better way to present may be in one final figure describing the overall model presented herein. In addition, I would like to see some of the data from SF1 moved into the main body of the text.

We appreciate the reviewer's comments. We reduced the number of schematics in the revision, including removing one from Fig. 2 and one from Fig. 7. We kept the ones in Fig. 1, 6 and 8 because they either provide critical information about the experimental design or a basis for

understanding the genetic perturbations, both of which make the results more understandable. To address the second issue, we now moved the data in SF1 and the relevant text to the Discussion section.

It would also be very interesting to see if the phosphate starvation also offered protection against other oxidative stressors such as tBOOH and how that effect the up/down regulation of Msn2/4 and Sch9.

We appreciate the reviewer's comment. This suggestion is addressed above.

The general execution of the manucript is of a high standard.

We appreciate the reviewer's positive comment.

Part II – Major Issues: Key Experiments Required for Acceptance
Please use this section to detail the key new experiments or modifications of existing experiments that should be absolutely required to validate study conclusions.

Generally, there should be no more than 3 such required experiments or major modifications for a "Major Revision" recommendation. If more than 3 experiments are necessary to validate the study conclusions, then you are encouraged to recommend "Reject".

Reviewer #1: No Major Issues

Reviewer #2: 1. One concern regarding the study is the lack of clarity on whether the induction of CTA1 alone is sufficient to confer acquired stress resistance (ASR) to H2O2. While the study indicates that CgCTA1 induction is required for ASR during phosphate starvation, it does not directly address whether CTA1 induction alone is adequate to establish the resistance phenotype. Further investigations, such as genetic manipulation experiments, are needed to determine the sufficiency of CTA1 induction in conferring ASR for H2O2.

We appreciate the reviewer's criticism and suggestions. We addressed the sufficiency issue in the main responses following the editor's summary.

2. Another concern relates to the functional characterization of CgMsn4 and CgSkn7 in the context of acquired resistance to H2O2 stress in C. glabrata. While the study suggests that these transcription factors contribute to CTA1 induction during phosphate starvation, it would be nice to show if CgMsn4 and CgSkn7 knock out strains display defects in the acquired resistance to H2O2 stress.

We appreciate the reviewer's question. We showed in the original manuscript that *msn4Δ* and *skn7Δ* both reduced *CTA1* induction, while the double deletion further reduced, but did not abolish *CTA1* induction. Based on this phenotype, we expect the single TFΔ mutants to have no or, at best, minor effects on ASR. Our CFU data supported this: compared to the wild type, neither *msn4Δ* nor *skn7Δ* significantly affected the ASR (See Fig. RR1 below). We decided not

to include the results in the manuscript both to limit the length and also because this result will be more informative once we identify the other TFs involved in the ASR.

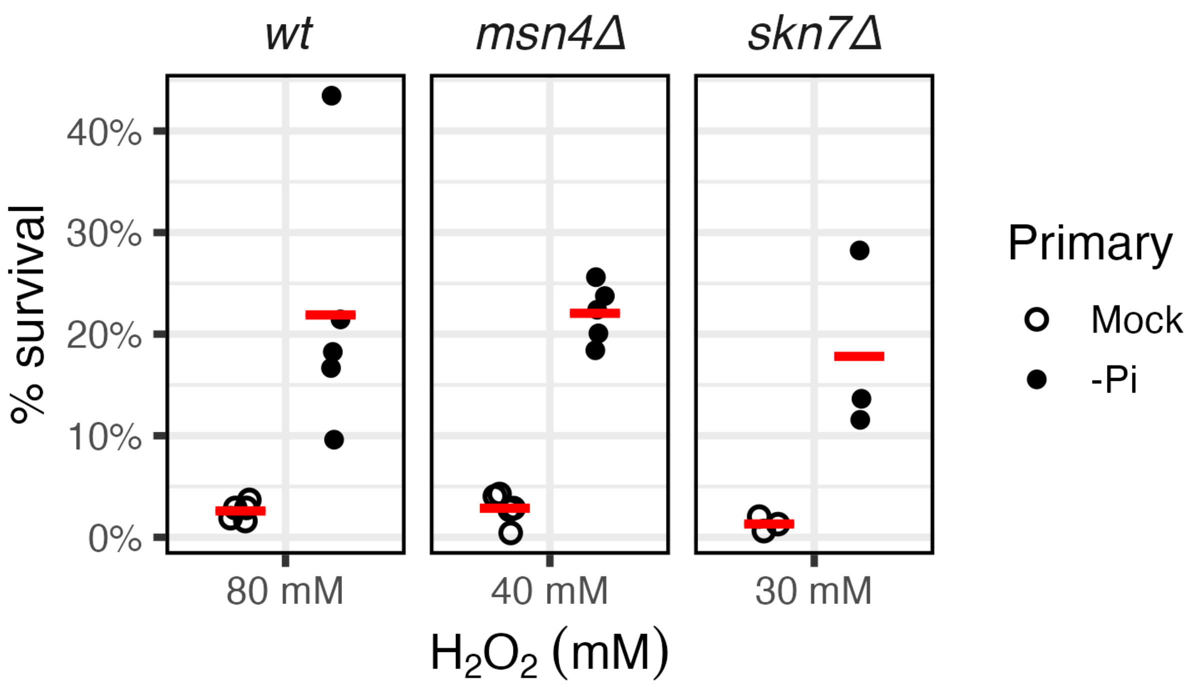

Fig. RR1 msn4Δ and skn7Δ have no detectable defects in ASR. We used the paired survival rates with primary stress (-Pi, solid dots) and without (Mock, open dots) to calculate the ASR-score. A Kruskal-Wallis Rank Sum test yielded a P-value of 0.31, suggesting there are no statistically significant differences among the three in their ASR-score.

3. Finally, the reviewer's concern is the lack of complemented strain. The inclusion of a few key complemented strains (such as CTA1) would strengthen the conclusions drawn from the knockout experiments. Additionally, it would be beneficial to mention the background of the parental strain used in the study.

We appreciate the reviewer's suggestions. We have now addressed the complement strain issue and described it in detail in the response to the editor's summary above. As for the second issue, we have now provided more detailed background genotypes for the parental strains used in this study in Table 1.

Reviewer #3: Screening on addtional oxidative stress chemicals post phophate starvation - is this limited to hydrogen peroxide only?

We appreciate the reviewer's suggestion and have addressed it in the responses following the editor's summary.

Part III – Minor Issues: Editorial and Data Presentation Modifications

Please use this section for editorial suggestions as well as relatively minor modifications of existing data that would enhance clarity.

Reviewer #1: • Although the conclusions drawn from panel 7A remain convincing, the non-phospho RPS6 antibody does not give a clear signal, and thus it's unclear whether quantitation (Fig. 7B) is suitable. Were both major bands treated as RPS6 during quantification?

We appreciate the reviewer's comment. Please see detailed response following the editor's summary.

• In supplementary figure 3, both panels, it appears that two different plate images are juxtaposed without space between them. This is misleading. There should be white space between the images from separate plates.

We thank the reviewer for this suggestion. In supplementary figure 3 (now Fig. S2), the two rows within each panel were cropped from the same plate image, although they were not adjacent on the plate. We agree that juxtaposing plate images, whether they were from the same or different plates, can be confusing. Therefore, we now add a spacer between the two rows. We also went through other figures and added spacers between plate images except when the images were contiguous from the same plate.

• It's odd that there is a figure callout to S1 in the introduction. Is there a way that this can be incorporated into the results section?

We appreciate the reviewer's comment. We now moved Fig. S1 to the Discussion section (Fig. S13). In the introduction, we only reference the other study to note that phosphate starvation is potentially relevant in the host, and leave the detailed discussion until later.

Reviewer #2: (No Response)

Reviewer #3: The figures are overly populated with schematics - one summary figure would be beneficial.

We appreciate the reviewer's comment and have removed some of the schematics. Please see our reply to Reviewer #2's summary above for more detail.

Reference cited

Hajdu T, Szabó K, Jakab Á, Pócsi I, Dombrádi V, Nagy P. 2021. Biophysical experiments reveal a protective role of protein phosphatase Z1 against oxidative damage of the cell membrane in Candida albicans. *Free Radic. Biol. Med.* 176:222–227.

Ikeda Y, Nakano M, Ihara H, Ito R, Taniguchi N, Fujii J. 2011. Different consequences of reactions with hydrogen peroxide and t-butyl hydroperoxide in the hyperoxidative inactivation of rat peroxiredoxin-4. *J. Biochem. (Tokyo)* 149:443–453.

Iwai K, Naganuma A, Kuge S. 2010. Peroxiredoxin Ahp1 acts as a receptor for alkylhydroperoxides to induce disulfide bond formation in the Cad1 transcription factor. *J. Biol. Chem.* 285:10597–10604.

Pichorner H, Jessner G, Ebermann R. 1993. tBOOH acts as a suicide substrate for catalase. *Arch. Biochem. Biophys.* 300:258–264.
